# Using Text Messaging to Assess Adolescents' Health Information Needs: An Ecological Momentary Assessment

**DOI:** 10.2196/jmir.2395

**Published:** 2013-03-06

**Authors:** Rebecca Schnall, Anastasia Okoniewski, Victoria Tiase, Alexander Low, Martha Rodriguez, Steven Kaplan

**Affiliations:** ^1^Columbia UniversitySchool of NursingNew York, NYUnited States; ^2^NewYork-Presbyterian HospitalNew York, NYUnited States

**Keywords:** text messaging, ecological momentary assessment, mobile health technology

## Abstract

**Background:**

Use of mobile technology has made a huge impact on communication, access, and information/resource delivery to adolescents. Mobile technology is frequently used by adolescents.

**Objective:**

The purpose of this study was to understand the health information needs of adolescents in the context of their everyday lives and to assess how they meet their information needs.

**Methods:**

We gave 60 adolescents smartphones with unlimited text messaging and data for 30 days. Each smartphone had applications related to asthma, obesity, human immunodeficiency virus, and diet preinstalled on the phone. We sent text messages 3 times per week and asked the following questions: (1) What questions did you have about your health today? (2) Where did you look for an answer (mobile device, mobile application, online, friend, book, or parent)? (3) Was your question answered and how? (4) Anything else?

**Results:**

Our participants ranged from 13-18 years of age, 37 (62%) participants were male and 22 (37%) were female. Of the 60 participants, 71% (42/60) participants identified themselves as Hispanic and 77% (46/60) were frequent users of mobile devices. We had a 90% (1935/2150) response rate to our text messages. Participants sent a total of 1935 text messages in response to the ecological momentary assessment questions. Adolescents sent a total of 421 text messages related to a health information needs, and 516 text messages related to the source of information to the answers of their questions, which were related to parents, friends, online, mobile apps, teachers, or coaches.

**Conclusions:**

Text messaging technology is a useful tool for assessing adolescents’ health behavior in real-time. Adolescents are willing to use text messaging to report their health information. Findings from this study contribute to the evidence base on addressing the health information needs of adolescents. In particular, attention should be paid to issues related to diet and exercise. These findings may be the harbinger for future obesity prevention programs for adolescents.

## Introduction

Understanding adolescents’ health behaviors is important because behavior that is unhealthy and/or risky, rather than infectious or chronic diseases, is the leading cause of morbidity among adolescents [1]. Moreover, adolescents’ health behaviors are still not well understood and have been under-studied [2]. We need a better understanding of adolescents’ health behaviors in the context of their daily living in order to develop interventions that target their health related activities. This is particularly important because behaviors and decision-making processes learned and habituated at a young age are likely to be more sustainable over time, and thus may have greater impact than attempting to change the behavior of adults [3].

One approach to addressing behavior change in adolescents is through the use of health information technology (HIT). Effective use of HIT for health communication purposes can facilitate clinical and consumer decision-making and build health skills and knowledge. For example, personal health records are an important and increasingly accepted tool for supporting patient-centered care, self-management, and effective use of health care resources [4]. There is also growing use of mobile health (mHealth) technologies, which are enabling individuals to manage their health. Use of mobile technology affords numerous advantages, including reduced memory bias, the ability to capture time-stamped data, and the potential for personalizing and tailoring information in real-time [5]. The ubiquitous nature of mobile technologies in daily life has created opportunities for applications that were not previously possible and allows for health resource monitoring and management [6]. Mobile technologies have the potential to transform health care by allowing clinicians to help patients address their health care needs in real-time [7]. For example, recent research has demonstrated the usefulness of text messaging on promoting the delivery of immunizations [8-13]. Nonetheless, these studies were limited to the use of text messaging by parents for managing their children and adolescents’ health. There has been a dearth of research on adolescents’ use of text messaging for managing their own health.

Mobile technology and text messaging are particularly appropriate for use in a study with adolescents since texting has become the preferred channel of communication between adolescents and their friends. In the United States, 2009, 77% of 12-17 year-olds owned cell phones, which increased from 45% in 2004 [14,15]. Cell phones have become indispensable tools in teen communication patterns [16]. In 2009, 88% of adolescent cell phone users used text-messaging, which was a large increase from the 51% in 2006 [14]. Adolescents send or receive an average of 3339 texts a month [17].

Using mobile phone technology and text messaging, we conducted a formative research study using a mixed methods approach to understand adolescents’ health information needs in the context of their everyday lives and to assess how adolescents meet their health information needs.

## Methods

### Sample Recruitment

Prior to the start of the study, approval was obtained from the Columbia University Institutional Review Board (IRB). IRB waiver of parental consent was obtained and participants signed assent forms prior to their participation in the study. Participants were recruited from a local public high school in Bronx, New York. Participants were recruited initially on-site, followed by snowball sampling, a non-probability sampling technique where existing study subjects recruit future subjects from among their friends, to identify additional participants until the desired number is recruited [18]. Participants were allowed to keep the mobile phone as compensation for their participation in the study.

### Data Collection

Prior to the start of the study, we collected demographic information and health related quality of life information from the participants using the 36-Item Short Form Health Survey (SF-36), selected from the Medical Outcome Study (MOS) inventory [19]. To evaluate health status in a valid and efficient way, Ware and colleagues developed the SF-36 to reduce questionnaire constraints faced in prior standardized health surveys [19-24]. As opposed to disease-specific measures, the SF-36 is a measure of generic health status or quality of life that includes both physical and mental health concepts. Following completion of the demographics and SF-36 forms, we used an ecological momentary assessment (EMA) to assess our participants’ health information needs in the context of their daily lives and determine how they met their needs. EMA is a sampling method developed to assess phenomena at the moment they occur in natural settings and takes place in participants’ naturalistic environments, supporting the ecological validity of this approach [25]. We used mobile smartphones and text messaging to conduct an EMA, which involved repeated sampling of subjects’ behaviors and experiences in real time, while the participants were in their natural environments [26].

**Figure 1:**
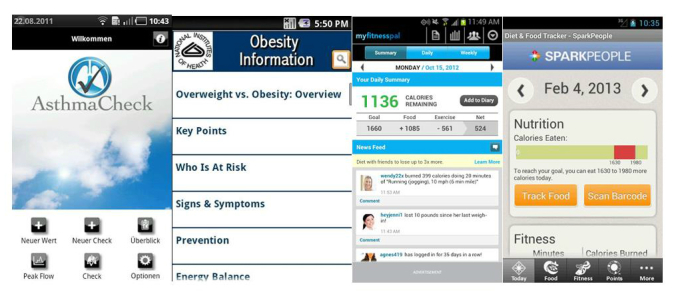
Screenshots of mobile apps which were downloaded on the smartphones prior to the EMA.

We gave 60 adolescents smartphones with unlimited text messaging and data access, as well as 600 voice minutes for 30 days. Each smartphone had applications related to asthma, HIV, obesity, diet, and exercise preinstalled on the phone. The applications which we installed were freely available on the Android and iPhone market and included: Myfitnesspal, Sparkpeople, NIH obesity, and Asthma Check (Figure 1). We used a hosted messaging gateway service to send text messages 3 times a week to ask adolescents the following questions: (1) What questions did you have about your health today? (2) Where did you look for an answer (mobile device/application, online, friend, book, or parent)? (3) Was your question answered and how? (4) Anything else?

Following 30 days of mobile phone use, we invited participants to attend a follow-up focus group session. The focus groups were held in a conference room at the Columbia University School of Nursing. Food appropriate for time of day was served and participants were compensated $20 for their time. We had a total of 4 sessions with 38 participants across all sessions.

### Data Analysis

#### Demographic Data

We used descriptive statistics to calculate the frequencies, means, and standard deviations of our sample population.

#### EMA Data

Two authors (RS and AO) independently coded each of the text message responses. The text message responses were divided into 3 groups based on the EMA questions: (1) health information needs, (2) information sources, and (3) information need resolved. Coding began after reading each of the text messages at least twice and highlighting the relevant ideas. Codes were created based on a line-by-line analysis. Data was summarized thematically through an iterative process by each author. After coding was completed, the authors met and discussed any discrepancies in their coding until reaching a consensus on an appropriate code.

#### Focus Group Data

Thematic analysis was used to code focus group data. Data was summarized thematically through an iterative process by the author after each focus group session. Similar procedures were used as in the text messaging coding.

## Results

### Sample

We had a total of 60 participants in our EMA study. One participant had her phone stolen but we replaced the phone. We turned off 2 cell phones before the end of the 30 days because those students used more than their allotted minutes. In this study, there were 37/60 (62%) males and 22/60 (37%) females with a mean age of 15.9 (SD 1.2) years. Our gender distribution was reflective of the gender makeup of the high school from which we recruited. There were 42/60 (70.0%) participants who described themselves as Hispanic. Participants reported the following racial categories: 16/60 (26.7%) black/African American, 1/60 (1.6%) white, 7/60 (11.7%) multi-racial, and 33/60 (55.0%) other. As would be expected for a population sample primarily composed of healthy adolescents, the response distributions for each of the 8 domains of the MOS SF-36 tended to be skewed in the direction of positive health. The mean physical functioning domain was 46.92 (SD 20.4), slightly below the national mean of 50 for adults.

Adolescents in our study had a lot of previous experience with mobile devices—35/60 (58.0%) had started using a mobile device more than 2 years ago, and 46/60 (76.7%) used their mobile device more than once per day. Usage of their mobile devices during the study period is illustrated in Table 1. There was no difference in mobile phone service use by gender (*χ* ^2^_4,57_=2.68, *P*=0.612).

**Table 1:** Mobile phone service usage over 30 days.

	Mean (SD)	Minimum	Maximum
Text	2514 (2751)	50	12,474
Data (megabytes)	4,848,708 (5,938,819)	33,461	28,235,106
Minutes	743 (1045)	0	4436

**Figure 2:**
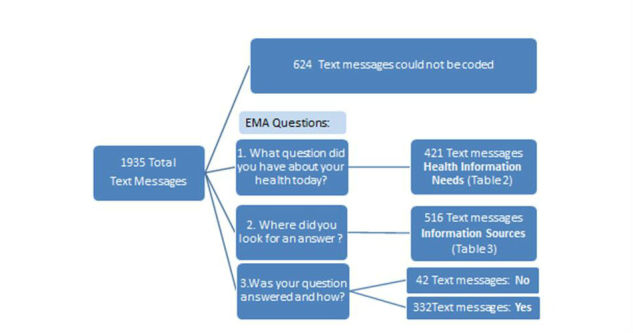
Flowchart of text message responses.

We had a 90% response rate to our text messages. Participants sent 1935 text messages over the study period (Figure 2). There were 624 text messages that could not be coded because they were irrelevant or provided too little detail to be well-understood (eg, “No nothing else”, “Tomorrow?”). Adolescents sent a total of 421 text messages related to a health information need. The health information needs are organized by themes in Table 2. Adolescents most often cited health information needs related to diet and exercise.

Adolescents sent 516 text messages related to the source of information to answer their question. Sources of information are categorized in Table 3. The most common source of information was parents (n=202). Following this information source, the Internet (n=200) was the most frequent. Of those who went online, 48/200 (24.0%) used Google as the main source for answering their health question.

In the final question of our EMA study, we sought to determine if adolescents found answers to their health questions. Participants reported that they had 421 health information needs and responded that their question was answered for 332 of their needs and not answered for 42 of their needs. Participants did not disclose whether 47 of their health information needs were met.

**Table 2:** Themes of health information needs and sample quotes.

Theme	n	Definition	Example
Diet/ Exercise	120	Nutrition, exercise, intake, calories, weight loss	*How much sugar should you have intake daily?* [EMA 19] *How come do dance everyday and still don’t loose [sic] weight?* [EMA 25]
Diagnosis/ clarification	117	Exploration of conditions, definition of medical terminology	*Can allergies go away?* [EMA 13] *Why am I the only one with asthma in my family?* [EMA 8]
Basic medical care	65	Triage of routine conditions, health status, treatment, or relief of condition	*How do I get rid of a headache?* [EMA 60] *What is the easiest way to deal with pimples?* [EMA 12]
Anatomy and physiology	33	Normal body functioning	*How much blood is in the human body?* [EMA 44] *How can I lower my heart rate?* [EMA 4]
Health promotion/ prevention	27	Way to prevent a condition or disease from occurring or reoccurring	*Can you really get diabetes by drinking soda?* [EMA 64] *Does smoking affect your sports life?* [EMA 16]
Reproductive health	23	Symptom identification of SDI’s, exposure, prevention, pregnancy	*My question is could you get HIV by kissing some body?* [EMA 20] *Does mirena diminish your possibilities of having kids later on in the future?* [EMA 8]
Psychological health	16	Psychological, psychiatric, or emotional issues	*My question today was what some common signs of depression are?* [EMA 48] *Can my emotional condition effect [sic] my physical condition?* [EMA 20]
Growth and development	11	Developmental norms, weight, height	*Is it healthy to weight [sic] 230 at 17?* [EMA 64] *If I am obese?* [EMA 68]
Emergency/ urgent medical care	9	First aid, bone/joint dislocations, asthma attack	*How you stop asthma attack?* [EMA 21] *What to do when you twist your ankle?* [EMA 1]

**Table 3:** Types of information sources and examples.

Type of resource	Total n	Example
Parent	202	*I asked my mom.* [EMA 62] *It was answer by my step father showing me different way to do it.* [EMA 19]
Online	200	*It was answers by online using a search site like Google.* [EMA 68] *Yes and by a medical Q and A website.* [EMA 63]
Friend	39	*I asked around to my friends.* [EMA 21]
Mobile device/ application	20	*I found answers on the information app on obesity on the phone.* [EMA 12]
Teacher/ coach	19	*I got the idea from my gym teacher.* [EMA 9]
Doctor/ nurse	14	*Doctor told me this.* [EMA 37]
Family	12	*I asked my sister for a answer.* [EMA 49]
Other	10	*Book at the library.* [EMA 19]

### Follow-up Focus Group Sessions

#### Overview

Of the 60 participants in our EMA study, 38 participated in the follow-up focus group sessions. During the focus group sessions, adolescents reported that they used their mobile phones for music, calendar, alarm, text messaging, pacer, games, navigation, camera, Tumbler, Google, Facebook, and YouTube. One adolescent said, “I just went on Facebook a lot and Google.” Another participant said, “If I have any health-related problems I usually just go on Google and it takes to me some doctor website, where real doctors answer the questions.” The websites that the adolescents reported were the same as those reported during their EMA, which triangulates our findings. Adolescents reported that they encountered multiple barriers when trying to use the mobile health applications that were installed on the phone. We identified the following barriers to mobile health application use: ease of use, readability, end user needs, and privacy.

#### Ease of Use

One participant stated, “it looked too complicated”. Another said, “I just didn’t understand it.” Participants also explained that they “didn’t want to read the whole thing, terms and agreements”. If the terms and agreements of an application were too long, then some participants “would just close the app rather than read it”.

#### Readability

The language contained within the application was also too difficult as one participant described, “They kept using big words.” To facilitate easy use of the applications, one participant recommended that we “dumb everything down”.

#### End User Needs

Participants reported that they did not like the apps because they were not tailored for adolescents’ use.

Honestly, I didn’t like the apps, because it’s like for older people like, I’m still a teenager, so I don’t want to be…boring.

One participant reminded the moderator that “teenagers think differently from other people”, and so the technology should be relevant to them, for example, “it can’t be like oh, has your Alzheimer’s been better?” Adolescents also emphasized the importance of the user interface and how they choose to use “the ones that looks cooler”. One participant explained that the health applications on the phone were “just the way [they looked], like it was boring”.

#### Privacy

Privacy is a consideration for adolescents when deciding to use mobile technology. Nearly all of our participants in our focus groups reported that they put a code on their phone to maintain privacy. Participants cited reasons such as “I want my stuff to be protected, like I don’t know who is going to go into my phone and do what with it.” One female expressed, “I don’t want my mom to see my messages.” Another participant said, “I don’t want anybody touching my stuff”, and the code would discourage others from using his phone.

In addition to putting codes on their phones, participants were concerned that the researchers could see what they were doing with their phones. One participant said, “I felt like there was a person sitting on a computer watching me.” Another participant said, “People don’t like being watched.” Finally, one adolescent said, “I thought that they were looking at our messages and stuff.”

## Discussion

Findings from our study demonstrated the usefulness of text messaging with health information as part of an EMA study with adolescents. EMA aims to minimize recall bias, maximize ecological validity, and allow study of behavior in real-world contexts. Since the mobile phone has become the favored communication tool for the majority of American teens [27], this tool offers promise for future investigation and data collection. While past studies have demonstrated the use of text messaging for delivering reminders to promote healthy behavior choices, these studies have been limited to parents’ use of this tool for managing their children’s health. In contrast this study provided preliminary evidence for the acceptability of the use of text messaging for adolescents managing their own health care. Moreover, our study demonstrated that adolescents are willing to use this technology and report on their own health questions in context of their daily lives. EMA with text messaging holds unique promise for future studies with adolescents who may be more resistant and unreliable for follow-up visits but may be willing to use text messaging in its place. In particular, our sample was unique in that they included many underserved and at-risk adolescents. This is noteworthy since developing these interventions for hard-to reach populations are critical and our study findings provide promise for the use of mHealth technology with these adolescents.

Since adolescents text frequently and have multiple health information needs, findings from our study suggested that development of health interventions using mobile technology has great promise. Nonetheless, while adolescents are interested in using applications and features on their phone, the language and interface needs to be tailored for adolescents’ use. Our study findings indicated that mHealth technology targeted for adolescent interventions must be designed specifically for adolescents, otherwise it is unlikely that they will be willing to use it. Particular attention should be paid to the user interface as well as developing tools, which must meet the literacy needs of underserved and high-risk adolescents [28-30]. Past research has demonstrated the importance of both of these issues in technology adoption, nonetheless, it is worthwhile to consider the needs of adolescents and tailor mobile apps to meet their literacy needs [31] and designing a user interface which is appealing to them [29,30,32].

Also notable were adolescents’ privacy concerns, which might be of interest to policymakers. With the passage of the Health Information Technology for Economic and Clinical Health (HITECH) act, personal health data will soon flow freely in electronic form with the provision that it is available securely and privately [33]. Nonetheless, it is important to consider adolescents’ views on the electronic transfer and viewing of their personal health information and their concerns about it. Concerns over the security of their personal health information may be a barrier to seeking health care services. 

While the results of our study indicated that adolescents’ health information needs are oftentimes met, it is important to educate adolescents who are heavy users of mobile technology and the Internet on how to verify their sources of information. As was evidenced by one of our participants who thought that “real doctors” answered her questions online, adolescents need to be educated so that they can be informed consumers of health information. Further research on how adolescents verify their sources of health information is warranted.

Of note were our participants’ most frequently reported questions about diet and exercise. While concerns over childhood obesity in our country continue to grow, our study indicated that our sample of ethnically diverse adolescents has heightened awareness and health information needs regarding issues related to obesity. Despite health information needs related to diet and exercise and past studies on this topic [34], a recent systematic review indicated that the outcomes of all of the existing obesity prevention programs across all age groups were modest and there were few replication studies of any program suggesting that the need for targeted interventions exist [35]. Three of the females in our follow-up focus groups reported that they used the preinstalled diet-tracking application, which was corroborated by data from the EMA text messages. They all reported that they used the application for only a few days because they forgot to continue using it or stopped their diet and did not want to feel guilty. Two participants recommended that we send push notifications to remind them to encourage continual use of the application in the future. These findings may be useful for informing the design of obesity prevention programs for adolescents, but further study is necessary.

Limitations to our study included a convenience sample that may not be representative of all ethnically diverse adolescents, particularly as they were recruited from one area of New York City. Even so, our sample had similar mobile technology use as other adolescents in the United States; past research has shown that 1 in 3 adolescents sends more than 100 text messages a day [16], while our participants sent an average of 84 (SD 91.72) texts per day. Another limitation of our study was the use of EMA. As with all self-report measures, there was no independent check on the veracity of the data, because all data were collected in the absence of the experimenter. In addition, since the browsing history of our participants was not assessed, responses to the text messages could be inaccurate owing to a number of factors including stigma. Finally, our technology was limited and did not have a decision tree associated with it to provide responses and feedback to our participants. This may have limited participants’ willingness to continue sharing their health information needs.

Findings from our study indicated the usefulness of text messaging technology as a tool for assessing adolescents’ health behavior in the context of their daily lives. Our study demonstrated that adolescents are willing to use text messaging to report their health information. Moreover, adolescents in our study provided useful information for the development of future health behavior tools using mobile technology.

## Abbreviations

EMA: ecological momentary assessment
HIT: health information technology
HITECH: Health Information Technology for Economic and Clinical Health
HIV: human immunodeficiency virus
IRB: Columbia University Institutional Review Board
mHealth: mobile health
MOS: Medical Outcome Study
SF-36: 36-Item Short Form Health Survey
